# Whole genome analysis of halotolerant and alkalotolerant plant growth-promoting rhizobacterium Klebsiella sp. D5A

**DOI:** 10.1038/srep26710

**Published:** 2016-05-24

**Authors:** Wuxing Liu, Qingling Wang, Jinyu Hou, Chen Tu, Yongming Luo, Peter Christie

**Affiliations:** 1 Key Laboratory of Soil Environment and Pollution Remediation, Institute of Soil Science, Chinese Academy of Sciences, Nanjing 210008, China; 2 Institute of Coastal Zone Research, Chinese Academy of Sciences, Yantai 264003, China

## Abstract

This research undertook the systematic analysis of the *Klebsiella* sp. D5A genome and identification of genes that contribute to plant growth-promoting (PGP) traits, especially genes related to salt tolerance and wide pH adaptability. The genome sequence of isolate D5A was obtained using an Illumina HiSeq 2000 sequencing system with average coverages of 174.7× and 200.1× using the paired-end and mate-pair sequencing, respectively. Predicted and annotated gene sequences were analyzed for similarity with the Kyoto Encyclopedia of Genes and Genomes (KEGG) enzyme database followed by assignment of each gene into the KEGG pathway charts. The results show that the *Klebsiella* sp. D5A genome has a total of 5,540,009 bp with 57.15% G + C content. PGP conferring genes such as indole-3-acetic acid (IAA) biosynthesis, phosphate solubilization, siderophore production, acetoin and 2,3-butanediol synthesis, and N_2_ fixation were determined. Moreover, genes putatively responsible for resistance to high salinity including glycine-betaine synthesis, trehalose synthesis and a number of osmoregulation receptors and transport systems were also observed in the D5A genome together with numerous genes that contribute to pH homeostasis. These genes reveal the genetic adaptation of D5A to versatile environmental conditions and the effectiveness of the isolate to serve as a plant growth stimulator.

Bacteria that efficiently colonize the rhizosphere and stimulate plant growth through direct or indirect mechanisms are referred to as plant growth promoting rhizobacteria (PGPR)^1^. In addition to possessing general plant growth promoting properties such as production of indole-3-acetic acid (IAA), siderophores^2^, 1-amino-cyclopropane-1-carboxylate (ACC) deaminase, hydrogen cyanate (HCN), nitrogenase^3^ and phosphate solubilization^4^, some PGPR also possess more environment specific plant growth promoting (PGP) traits such as heavy metal detoxifying activity^5^, salinity tolerance^6^, and biological control of phytopathogens and insects^7^.

PGPR have become of interest as inoculants for phytoremediation because of their diverse plant growth promoting capabilities^8^. Systematic analysis of whole genome data and the identification of genes that contribute to the beneficial activity of PGPR will aid our understanding of the molecular mechanisms of many bacterial species and also help in the development of PGPR assisted phytoremediation technology. Next generation sequencing technologies (NGS) have enabled whole genome sequencing of bacteria and other organisms^9^,^10^,^11^. NGS have recently been employed to study the genomes of several PGPR such as *Pseudomonas* sp.^12^, *Bacillus* sp.^13^, and *Paenibacillus polymyxa*^14^.

In 2013 a plant growth promoting bacterium, *Klebsiella* sp. D5A, was isolated from the rhizosphere soil of tall fescue (*Testuca arundinacea* L.) growing in a soil contaminated with oil and the PGP traits and environmental tolerance of this strain were quantified. The bacterium produced IAA, solubilized phosphate, synthesized sideropheres, and also had a strong ability to adapt to a high saline-alkaline environment and wide range of soil pH (4–10)^15^. Pot experiments indicate that inoculation with this isolate promoted the growth of host plants in a petroleum-contaminated saline-alkaline soil and enhanced phytoremediation efficiency. *Klebsiella* sp. is likely to be an effective PGPR due to some members being endophytic nitrogen-fixing bacteria^16^,^17^,^18^,^19^,^20^. Some *Klebsiella* genomes have now been reported^18^,^20^,^21^,^22^ but most investigations have focused on their pathogenic or N-fixation genes. By contrast, the genes that contribute to the beneficial activity of PGPR and especially salt tolerance and pH adaptability remain to be studied in detail.

Here, we report the complete genome sequence of *Klebsiella* sp. D5A to help reveal the complex biological mechanisms of D5A as a PGPR. The genomic analysis in this study also includes comparison of the PGP traits to four closely related and representative PGPR strains that have been studied previously, namely *K. variicola* 342^18^ (originally misclassified as *K. pneumoniae* and then clustered with *K. variicola* by Garza-Ramos *et al*.)^23^, *K. variicola* At-22^19^,^20^ and *K. variicola* DX120^20^, *K. pneumoniae* MGH78578^18^. The genome analysis will provide a fundamental basis for future studies towards fully understanding the functions of this organism. Furthermore, comparisons among the completely sequenced *Klebsiella* genomes will help to offer new insights into evolutionary changes in *Klebsiella* spp. and highlight the genes that may contribute to their plant growth-promoting properties.

## Results

### General genome features of *Klebsiella* sp. D5A

**Figure 1:**
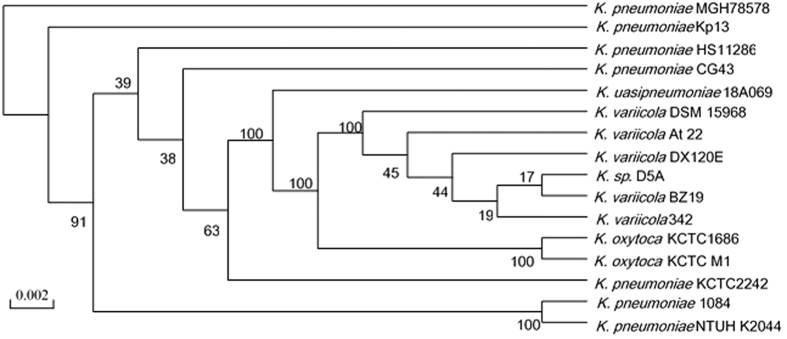
Phylogenetic tree of 16 different *Klebsiella* species based on aligned concatenated sequences of 45 orthologous genes using the bootstrap method. Numbers on nodes represent percentages of individual trees containing that relationship. The scale bar corresponds to the number of substitutions per site.

The genome of *Klebsiella* sp. D5A has a total of 5,540,009 bp with an average G + C content of 57.15% (Table 1). The genome contains 4999 predicted CDSs (coding sequence) with an average length of 944 bp. Among these CDSs, 4339 (86.8%) genes were classified into COG (Clusters of Orthologous Groups of proteins) families composed of 21 categories (Table 2). Coding regions cover 85.2% of the whole genome. Biological roles were assigned to 4129 (82.6%) genes of the predicted CDS based on similarity searches with the String and Nr database. The remaining 870 (17.4%) coding sequences were classified as proteins with unknown or hypothetical function. A total of four rRNAs comprising two 5S rRNAs, a single 16S rRNA, and a single 23S rRNA togther with 73 tRNA genes representing 37 amino acids were identified in the D5A genome. We established a phylogenetic tree of 16 completely sequenced *Klebsiella* based on 45 conserved genes. The tree shows that *Klebsiella* sp. D5A is most closely relate to *K. variicola* (Fig. 1).

### Genes related to plant growth promotion traits of *Klebsiella* sp. D5A

**Figure 2:**
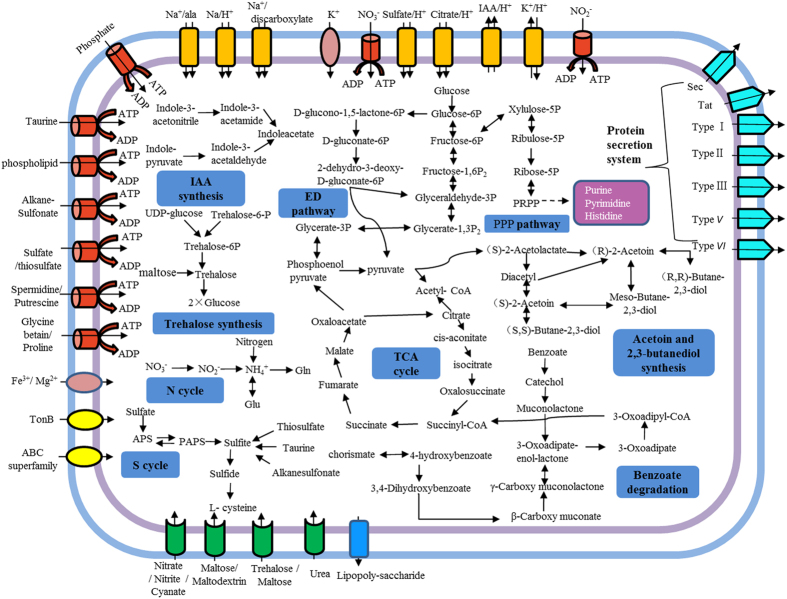
Schematic overview of metabolic pathways and transport systems of *Klebsiella* sp. D5A. The depicted pathways were predicted based on the genomic data of *Klebsiella* sp. D5A analyzed by Glimmer. Individual pathways are denoted by single headed arrows and reversible pathways are denoted by double-headed arrows.

We identified genes in the D5A genome attributable to the production of IAA, solubilization of phosphate, synthesis of sideropheres, acetoin and 2,3-butanediol, suppression of pathogenic fungi, resistance to oxidative stress, and ability to break down toxic compounds and other abiotic stresses (Table S1). Our previous study shows that *Klebsiella* sp. D5A actively produces 112 mg L^−1^ IAA (Table 3) and IAA production is unaffected by pH values between 4 and 10^15^. Here, two proposed IAA biosynthesis pathways, indole-3-acetonitrile (IAN) and indole-3-pyruvate (IPyA) pathways are identified in the genome of D5A (Fig. 2) and four genes might be involved (Table S1). In the IAN pathway IAN can first be converted to indole-3-acetamide (IAM) by nitrile hydratase and then IAM is converted to IAA by amidase. In the IPyA pathway indole-3-pyruvate (IPyA) is converted to indole-3-acetaldehyde (IAAld) by indolepyruvate decarboxylase and then to IAA by aldehyde dehydrogenase. A search of four sequenced *Klebsiella* genomes (Table S2) for D5A-like IAA pathway associated genes reveals the presence of three orthologous genes in *K. variicola* 342, *K. variicola* At-22 and *K. variicola* DX120 compared to D5A. They lack a gene coding for aldehyde dehydrogenase. All of these genes responsible for IAA synthesis were absent from *K. pneumoniae* MGH78578.

Gluconic acid (GA) is recognized as one of the major organic acids in most bacteria responsible for the solubilization of mineral phosphates. The synthesis of GA is catalyzed by glucose dehydrogenase (GDH) and its co-factor pyrrolo-quinolone quinine (PQQ)^24^,^25^. As Table 3 shows, D5A can solubilize 131 mg L^−1^ phosphates. Accordingly, the D5A genome possesses genes encoding GDH activity and carries the redox co-factor pqq genes including *pqqBCDEF* while lacking the gene *pqqA.* Toyama and Lidstrom^26^ have reported that the enzyme encoded by *pqqA* is not essential for biosynthesis of PQQ in *Methylobacterium*. In addition, inorganic phosphate uptake in D5A may be promoted by one low-affinity phosphate transport system, *PitA*, and two high-affinity phosphate transport systems, *PstBACS* and *PhnCDE2E1* (Table S1).

D5A carries genes coding for the synthesis of siderophores, such as *entABECFD* which are responsible for the conversion of chorismic acid to enterobactin and for the transport of this siderophore (*entS*) which is found ubiquitously among enterobacteria (Table S1). However, the genes involved in pyoverdine synthesis, the main class of siderophore, are absent from D5A. Furthermore, bacteria may also heterologously adopt siderophores produced by other organism via various siderophore receptors^27^. *Klebsiella* sp. D5A encoded 12 copies of genes for siderophore receptors including five TonB-dependent outer-membrane receptors, three putative ferric enterobactin receptors (*fepA*), a catecholate siderophore receptor (*fiu*), a ferric aerobactin receptor, a ferrioxamine receptor (*foxA*), a ferrichrome outer membrane receptor, and the ferric uptake regulator (*fur*) (Table S1). Furthermore, 43 additional ORFs (open reading frames) that encode iron transport such as the ferrous iron transporter which synthesizes proteins involved in Fe^2+^ capture and the *sitABCD* system involved in the transport of divalent cations such as Mn^2+^ and Fe^2+^ 22 were also determined in the D5A genome (Table S1). These indicate that although strain D5A cannot synthesis numerous sideropheres, it can heterologously obtain siderophores produced by other soil bacteria.

In addition to the above PGP traits, two growth-promoting volatile organic compounds (VOCs), acetoin and 2,3-butanediol, were reported to promote plant growth by stimulating root formation^28^ and increasing systemic disease resistance^29^ and drought tolerance^30^ in some other very efficient PGPR. Genes encoding enzymes including acetolactate synthase, acetolactate decarboxylase, and acetoin reductase (Table S1) which are involved in acetoin and 2,3-butanediol synthesis, were detected in the genome of D5A and the synthetic pathway is shown in Fig. 2. First, two pyruvate molecules are condensed into acetolactate catalyzed by acetolactate synthase, and then acetolactate is converted to acetoin by acetolactate decarboxylase and finally acetoin is converted to 2,3-butanediol catalyzed by acetoin reductase. When the genomes of the other four *Klebsiella* isolates were examined for acetion and 2,3-butanediol synthesis, partial or incomplete coding genes were observed while the synthesis of the two VOCs was not influenced.

It has been reported that PGPR may produce compounds such as phenazine and 4-hydroxybenzoate which function as antibiotics and suppress plant pathogenic microbes^12^,^31^. *UbiC*, involved in 4-hydroxybenzoate synthesis, and *phzF*, involved in phenazine synthesis, were identified in the D5A genome. Moreover, a homologue of the gene coding for chitinase enzyme was identified here which can dissolve the cell walls of pathogenic fungal and insect pests^31^,^32^. In addition to these we found the genes *gabD* and *gabT* which are responsible for the production of pest/disease inhibiting γ-aminobutyric acid (GABA)^31^ in the genome. A search for these genes in four *Klebsiella* genomes found that they were present in the four bacteria except that genes coding for chitinase enzyme were absent from *K. variicola* 342. This suggests that the synthesis of the three antimicrobial compounds is a widespread pathway in *Klebsiella* spp.

Furthermore, some other PGPR fitness conferring genes were also detected in the D5A genome. For example, the genes *speA, speB* and *speE* which encode for, respectively, arginine decarboxylase, agmatinase, and spermidine synthase may lead to the transformation of amino acids to PGP substances^33^. Moreover, the D5A genome encodes numerous proteins to protect the cell from oxidative stress: eight peroxidases, three catalases, four superoxide dismutases, three hydroperoxide reductases and 13 glutathione S-transferases (Table S1).

Production of cold-shock and heat-shock proteins by microoganisms can help their survival in harsh environments. The D5A genome carries the heat-shock protein genes *dnaJ, dnaK, groEL, groES, htpG*, and *htpX* and the cold-shock protein gene *cspA* (Table S1). Moreover, the gene coding for the enzyme rhodanese was found in the D5A genome and this is responsible for the detoxification of cyanide in organisms (Table S1). Cyanide is a potent cytotoxin which is produced by the hydrolysis of plant cyanogenic glycosides and may inhibit the cytochrome oxidases in the mitochondrial electron transport chain^34^.

### Nitrogen fixation

*Klebsiella* sp. D5A is able to grow on nitrogen-free medium (Table 3) and this indicates that the strain is able to fix atmospheric nitrogen. Nitrogenase is the enzyme central to nitrogen fixation and it consists of Fe-protein encoded by *nifH* and MoFe-protein encoded by *nifDK*. Full assembly of the nitrogenase complex needs the products of at least twelve *nif* genes, especially for the processing of catalytic stability and nitrogenase metalloclusters (*nifMZ, nifUS*, and *nifW*) and for synthesis of a particular molybdenum cofactor (FeMo-co)^35^. The D5A genome contains all the above *nif* genes together with the *NifA* and *NifL* genes which are the positive/negative regulatory proteins for *nif* genes^36^. The *rnfABCDEG* operon, which encodes a membrane-bound protein complex related to electron transport to nitrogenase^37^, is also found in the D5A genome (Table S3). In contrast, comparative genomic analysis shows that key genes associated with nitrogen fixation including nitrogenase are absent from *Klebsiella* sp. MGH78578. It is therefore presumed that MGH78578 cannot fix nitrogen.

### Genes putatively involved in salt tolerance

As Table 3 shows, D5A can grow well in 0–12% NaCl. Analysis of the genome reveals that strain D5A has a number of genes related to salt tolerance. For example, the key genes *betA* and *betB* for glycine-betaine synthesis that respectively encode choline dehydrogenase and betaine aldehyde dehydrogenase were found in D5A. They are considered to be the most effective genes responsible for salt tolerance^38^. In addition, trehalose can act as an osmoprotectant under environmental stresses such as high salt or drought, low temperature or osmotic stress in many organisms^12^. Trehalose accumulates in transgenic rice and enhances plant abiotic stress tolerance^39^. So far five trehalose biosynthetic pathways have been found in bacteria including treS, otsA/otsB, treP, treT and treY/treZ^40^. Here, two trehalose biosynthesis pathways, treS and otsA/otsB, were identified in the D5A genome. In the treS pathway maltose is converted to trehalose by trehalose synthase (treS). In the otsA/otsB pathway both glucose-6-phosphate and UDP-glucose can synthesize trehalose-6-phosphate catalyzed by trehalose-6-phosphate synthase (*otsA*) activity. Trehalose-6-phosphate is then formed from trehalose catalyzed by trehalose-6-phosphate phosphatase (*otsB*) activity. Eventually, trehalose may be hydrolyzed by trehalase (*treA, treF*) with the generation of two glucose molecules (Fig. 2, Table S1). This pathway has been recognized as a universal pathway present in microorganisms and it may further contribute to survival under harsh environmental conditions^12^.

Moreover, a number of osmoregulation receptors and transport systems were determined in the D5A genome. These genes can encode up to 24 two component systems (TCSs), among which 16 TCSs can be functionally assigned based on the KEGG database (Table S4). Of those 16 assigned TCSs, seven belong to the OmpR family, five to the NarL family, one to the LytT family, one to the NtrC family and two to the CitB family. The eight remaining TCS genes are annotated as sensor histidine kinase (Table S4). For example, the KdpD/KdpE TCSs that belong to the OmpR family have genes responsible for testing hyperosmotic stress and regulating the expression of genes in cell wall synthesis and for the accumulation of compatible solutes^41^. They may also activate the expression of the *kdp* operon which encodes the high-affinity K^+^ uptake system (Kdp) in response to high salt stress^42^. In addition, genes encoding transport systems such as K^+^ transport systems for K^+^ accumulation^43^ and Na^+^/H^+^ antiporters (*nha*) for importing H^+^ and pumping out Na^+^ 44 have also been found to resist hyperosmotic stress in the genome of D5A (Table S5).

### Response of D5A to pH and genes putatively involved in wide pH adaptation

D5A was isolated from a saline-alkali oilfield which had a pH of 9.7 and it was adapted to a wide range of pH conditions as its growth was unaffected by pH values between 4 and 10. By contrast, common neutralophilic bacteria grow over a narrow range of external pH values (5.5–9.0) and maintain a near-neutral cytoplasmic pH that lies within a pH range of 7.5–7.7^45^,^46^. Hence, they are able to acidify or alkalize the cytoplasm relative to the external environment to meet pH challenges by direct active uptake or efflux of protons. For instance, under conditions of acid challenge the gene *kdpABC* in the D5A genome that encodes the high-affinity K^+^ transport system can be activated by *kdpD/kdpE*. This may cause an inside-positive membrane potential by activating K^+^ influx and thus avert the ingress of protons^47^. Cytoplasmic pH has also been reported to be maintained by the metabolism of proton buffer molecules such as phosphate uptake (*pstSCAB*) and the expression of amino acid decarboxylase like arginine (*speA*), aspartate (*panD*) and lysine (*cadA*) decarboxylation, all of which were found in the D5A genome (Table S6). Moreover, some genes responsible for acid resistance occur in acid mine drainage environments as reported by Guazzaroni *et al*.^48^ including the nucleic acid-binding proteins of Hu (*hupA, hupB*), ClpXP (*clpX, clpP*) proteins, and Dps (*dps*), and the transcriptional repressor LexA (*lexA*) have also been found in the D5A genome. The genes encoding urease (*ureABC*) and urease accessory protein (*ureEFGDH*) that increases ammonia production are present in D5A and have an acid resistance ability^49^,^50^. In addition, several protective proteins including *GroEL, DnaK*, and *HdeB* chaperone that might be induced under acid stress^48^ were found in the D5A genome (Table S6).

For pH homeostasis under alkaline stress, inward transport of protons is an important adaptation mechanism that usually involves the activation of key cation/proton antiporters. A total of eight cation/proton antiporters exist in the D5A genome, including Na^+^/H^+^ antiporters, K^+^/H^+^ antiporters, Ca^2+^/H^+^ antiporters and some combinations of these cytoplasmic cations that exchange cations for external H^+^ moving inward (Table S6). A multidrug transporter gene (*mdfA*) was identified here which was earlier described as activating the outflow of numerous drug substrates in exchange for H^+^ and also catalyzing K^+^/H^+^ antiporter and Na^+^/H^+^ antiporter activity. This might support bacterial growth at pH > 9. Furthermore, it is well known that the genes encoding F_1_F_0_-ATP synthase which imports protons during ATP synthase activity will be induced under alkaline conditions. The D5A genome has an *atp* operon with the expected eight ATP synthase protein-encoding genes and an *atpI* gene whose protein product contributes to the stability and assembly of the synthase. Identification of the *cls* gene in D5A that encodes membrane cardiolipin in alkaliphiles will support oxidative phosphorylation by facilitating rapid proton transport along the membrane surface. In addition, expression of genes coding for periplasmic proteins such as OmpA, MalE and OmpX porins in the D5A genome likely leads to metabolic modes that are adapted to high pH. Some amino acid catabolism enzymes such as serine deaminase (*sdaA*) may also function at high pH (Table S6).

### Degradation of aromatic compounds

D5A was isolated from an oil-polluted soil and can therefore potentially be adopted in oilfield bioremediation. Aromatic compounds have been recognized as the most recalcitrant and abundant pollutants in oilfields. Numerous genes associated with aromatic compounds have been determined in the D5A genome. For example the genes involved in 3-hydroxyphenylpropionate (3-HPP) catabolism including the *mhpRABCDFET* operon were identified here (Table S7). In addition, the D5A genome also possesses a complete β-ketoadipate pathway through the protocatechuate and catechol routes for further degradation of the ring cleavage products to TCA cycle intermediates (Fig. 2) which are derived from 4-hydroxybenzoate and benzoate separately. More than 19 genes involved in the protocatechuate (*pca* genes) and catechol (*cat* genes) branches of the β-ketoadipate catabolism pathway were identified in D5A (Table S7). Protocatechuate is recognized as one of the key intermediates during the catabolism of various aromatic compounds^51^. This pathway is considered to be one of the key routes for the degradation of aromatic compounds and is similar to those found in other *Klebsiella* genomes. This indicates that D5A has a broad potential for the degradation of aromatic compounds.

### Central metabolic pathways

A schematic summary of the metabolic patterns in *Klebsiella* sp. D5A is shown in Fig. 2. The genome of *Klebsiella* sp. D5A shows that it carries genes consistent with the ability to survive in the soil environment and in plant rhizospheres. The genome also contains a complete carbohydrate metabolism pathway including glycolysis/gluconeogenesis, the tricarboxylic acid (TCA) cycle, pyruvate metabolism, and the pentose phosphate (PPP) and Entner-Doudoroff (ED) pathways.

Sulfur metabolism in D5A includes mineralization of organic sulfonates and assimilation of inorganic sulfate (Fig. 2). A total of 17 ORFs encoding inorganic thiosulfate or sulfate transporter and convert-related genes were present in the D5A genome (Table S8). Alkyl/aryl-sulfonates are considered to be key components of the sulfur present in agricultural soils^34^. In the genome of D5A, extracellular alkanesulfonates are first transported into the cell by aliphatic sulfonate ABC transport (*ssuABC*) and then catalyzed by alkanesulfonate monooxygenase (*ssuD*) and an NADPH-dependent FMN reductase (*ssuE*). Moreover, the D5A genome has four genes encoding taurine transporter-related proteins, *tauABCD* (Table S8). In addition, a transcriptional regulator coding gene *cysB* which can mediate global sulfur regulation in many gram-negative bacteria was identified in the D5A genome. This activates the transcription of cysteine synthesis genes under conditions of sulfur limitation^52^. Recently, hydrogen sulfide (H_2_S) produced by PGPR has been reported to increase seed germination and promote the growth of the plant roots that they colonize. The genes *cysCIJN* that are responsible for H_2_S biosynthesis as reported by Dooley *et al*.^53^ have been found in the D5A genome.

### Secretion systems

D5A has seven potential protein secretion systems including Types I, II, III, V and VI, Tat (twin arginine translocation), and Sec (general secretory pathway) (Table S9). The Sec and Tat systems are the two widespread systems for transport across the cytoplasmic membrane. D5A has both of the secretion systems. In contrast to strain D5A, the other four *Klebsiella* genomes all lack the genes encoded for Types III and V but only *K. variicola* 342 possess the genes encoded for Type IV.

## Discussion

In this study we report the whole genome sequencing and analysis of *Klebsiella* sp. D5A isolated from the rhizosphere soil of tall fescue growing in an oil-contaminated soil. The genome data of this strain support and extend various laboratory observations that have been reported in our previous study. Liu *et al*.^15^ found that *Klebsiella* sp. D5A exerts beneficial effects on plant growth as it may promote tall fescue germination rate, root length and shoot height. Consistent with the PGP properties that have been referred in our earlier study, we found genes attributable to IAA production, phosphate solubilization and siderophore synthesis. In addition, other PGP trait coding genes including acetoin and 2,3-butanediol synthesis, putrescine and spermidine synthesis and some other PGPR fitness conferring genes have also been found in the D5A genome. Genes with similar functions in other PGPR have been previously been reported from other studies^12^,^34^,^54^. In addition to their direct plant growth promoting abilities PGPR also support plant growth indirectly by suppressing pathogens^55^. In the D5A genome we have identified many genes that are well known to be responsible for the production of antimicrobial compounds such as 4-hydroxybenzoate, phenazine, chitinase and GABA. The genome also encodes enzymes including catalases, peroxidases, glutathione transferases and superoxide dismutases, all of which are responsible for resisting oxidative stresses in plants. Genome sequence analysis also shows that D5A has many genes corresponding to Sec, Tat and Types I, II, III, V, and VI (Table S9). Some earlier studies have reported that the presence of Type I–VI and Sec secretion systems in the rhizobacteria *Variovorax paradoxus* and *Pseudomonas fluorescens* may function in promoting plant growth^12^,^34^,^56^ and also help in their rhizosphere colonization^57^.

A fundamental role of the *Klebsiella* genome is its capacity to fix nitrogen^19^,^20^,^58^. 20 proposed *nif*-specific genes which are related to nitrogenase were all found in the D5A genome. In addition, sulfur is recognized as an essential nutrient for plant growth and is closely connected with stress tolerance in plants^59^. Generally, plants acquire sulfur from soils and rely on the mobilization of this sulfur for assimilation by plants as determined by the soil microbial community^60^. In the D5A genome we have found genes related to H_2_S synthesis and they may be an important sulfur source for plant growth^31^. We have also identified genes in the D5A genome that are involved in the degradation of aromatic compounds, suggesting a function in the catabolism of some organic compounds. The comparative genomics analysis of some PGP traits with four other representative *Klebsiella* PGPR strains reveals some conserved genes among the different *Klebsiella* species, such as IAA, solubilization of phosphates, scetoin and 2,3-butanediol synthesis and production of antimicrobial compounds, except for some strain-specific genes differentiated in each strain. This provides clues as to the characteristics common to *Klebsiella* PGPR.

Liu *et al*.^15^ found that strain D5A was capable of surviving and growing well under a wide range of NaCl concentrations which may help it establish well under harsh environmental conditions. The genes involved in betaine, trehalose, acetoin and 2,3-butanediol biosynthesis were detected in the D5A genome and these have been implicated in the survival of some microorganisms under saline or osmotic stress conditions^38^. In addition, many genes involved in osmosensing and regulation genes that belong to TCSs, as well as Na^+^/H^+^ antiporters (*nha*) for importing H^+^ and pumping out Na^+^ 44 were present in the D5A genome. Furthermore, the existence of K^+^ transporters may help the K^+^ influx that is regulated by KdpD/KdpE TCS and the possible accumulation of K^+^ inside the cells may be activated to resist osmotic pressure^61^. The coefficient of TCSs and related transporters might alter membrane permeability and activate the expression of osmoresponsive genes^62^. In addition, the presence of genes that regulate the production of cold-shock and heat-shock proteins and osmoregulants in the D5A genome may also help in adaptation to harsh environments for survival.

Strain D5A was isolated from a highly alkaline soil (pH 9.7) and previous experiments have indicated that it can adapt to a wide range of pH values as its growth was unaffected by pH levels between 4 and 10^15^. It is clear that adaptation to a wide range of environmental pH requires a robust internal pH homeostatic system for bacteria, thus maintaining a near-neutral cytoplasmic pH that is suitable for structural integrity and optimum functionality of the cytoplasmic proteins^45^,^63^. Numerous adaptive strategies are deployed for pH homeostasis under acid/alkaline pressures including variation in transport and metabolic patterns. The primary strategy for bacterial pH homeostasis is using the transporters that directly regulate the uptake or outflow of protons. For example, when D5A was exposed to acidic conditions the *kdpABC* genes in D5A that encode the ABC high-affinity potassium transport system were regulated by *kdpD*/*kdpE* and generated an inside-positive membrane potential through active influx of K^+^ to partially deflect the inward flow of protons whereas the expression of key cation/proton transporters that extrude cytoplasmic Na^+^, K^+^ and Ca^2+^ in exchange for H^+^ would be active and function under alkaline conditions. Among these cation/proton transporters, Na^+^/H^+^ antiporters (*nhaA*) are recognized as having the predominant role in alkaline pH homeostasis as the stoichiometry for *nhaA* is 2H^+^/1Na^+^. However, *nhaA* is a model of pH-regulated antiporter for pH homeostasis and is unable to support the growth of *Escherichia coli* at pH > 9 while *mdfA* can do so^46^. The gene *mdfA* that encodes multidrug translocase was found in the D5A genome. The expression of F_1_F_0_-ATP synthase that carries protons into the cell during ATP synthesis will decrease under acidic conditions and the hydrolysis of F_1_F_0_-ATPase that facilitates ATP-dependent H^+^ outflow will be upregulated^64^.

A secondary strategy for pH homeostasis is the remodeling of metabolic patterns that regulate proton generation or consumption by metabolic enzymes^45^. In the D5A genome, genes encoding amino acid decarboxylases such as arginine (*speA*), aspartate (*panD*) and lysine (*cadA*) decarboxylation would help the strain survive by acid tolerance by consuming protons and thus lead to decreased concentrations of free protons in the cytoplasm^64^. In contrast, challenges by alkaline conditions will lead to activation of amino acid deaminases such as serine deaminase (*sdaA*) determined in the D5A genome^65^. Deaminases can provide an acid-generating mechanism that is adaptive to alkaline conditions as decarboxylases promote alkalization that is adaptive to acid enviroments^46^. Moreover, genes encoding ClpXP protease, urease, the transcriptional repressor LexA and nucleic acid-binding proteins such as an RNA-binding protein, HU and Dps, that are found in the D5A genome (Table S6) have been reported to be able to expand the capability of bacteria to survive under severe acid stress^48^. The existence of these genes carried by the D5A genome provides a fundamental function for pH homeostasis, while the detailed elucidation of how they function or contribute to pH, cation or osmotic homeostasis requires further transcription studies.

## Conclusions

The *Klebsiella* sp. D5A genome has a total of 5,540,009 bp with a 57.15% G+C content. Whole-genome comparison with 15 other completely sequenced *Klebsiella* strains reveals that D5A belongs to the *K. variicola* group. As confirmed from previous reports and supported by the genome analysis in the present study, *Klebsiella* sp. D5A is demonstrated to act as a PGPR as the genes potentially involved in plant growth promotion such as indole-3-acetic acid (IAA) biosynthesis, phosphate solubilization, siderophore production, acetoin and 2,3-butanediol synthesis, N_2_ fixation, chitinase, phenazine, 4-hydroxybenzoate, and H_2_S synthesis, and some other PGPR conferring genes were found. Comparative genomic analysis of four other representative PGPR with D5A reveals some conserved regions indicating common PGP characteristics among these *Klebsiella* PGPR. The *Klebsiella* sp. D5A genome also contains sets of catabolic genes involved in the degradation of aromatic compounds. Moreover, genes that contribute to the high salinity resistance of D5A were also observed including glycine-betaine synthesis, trehalose synthesis and a number of osmoregulation receptors and transport system coding genes. In addition, the strain grew well in the pH range 4–10 with the putative genes responsible for pH homeostasis but detailed elucidation of how these genes are regulated and function requires further transcription studies.

## Materials and Methods

### PGPR strain

*Klebsiella* sp. D5A is a previously studied gram-negative PGPR isolated from the roots of tall fescue. The 16S rRNA gene sequence of D5A exhibits the highest (99.5%) sequence similarity to *Klebsiella variicola* (HQ259961). Details of the plant growth promotion properties (IAA production, phosphate solubilization, siderophore production) and environmental tolerance (pH, NaCl, Na_2_CO_3_ tolerance level) of *Klebsiella* sp. D5A are shown in Table 3 15. Additionally, the nitrogen fixation property was tested according to Wu *et al*.^66^. Given the beneficial attributes of D5A, we chose to characterize them further at the genomic level.

### Bacterial growth and DNA extraction

A single colony of *Klebsiella* sp. D5A grown on ADF agar medium^67^ was inoculated into 100 mL of LB medium (10 g tryptone, 5 g yeast extract and 10 g NaCl per liter) and shaken at 150 rpm at 30 °C for 48 h. Bacterial cells were collected by centrifugation and the genomic DNA was extracted with a Fast DNA SPIN kit (MP Biomedicals, Solon, OH) according to the manufacturer’s instructions and the DNA was checked on agarose gel.

### Genome sequencing and annotation

The genome sequence of *Klebsiella* sp. D5A was determined by Shanghai Majorbio Bio-Pharm Technology Co., Ltd. (Shanghai, China) using the HiSeq 2000 sequencing platform (Illumina Inc., San Diego, CA). The prepared DNA samples were used to construct the pair-end and mate-pair sequencing libraries. The average fragment sizes for the pair-end and mate-pair libraries were 300 and 3,000 bp, respectively. The read length was 101 bp. Low quality sequence data were cut and then the reads were assembled using the SOAPdenove v2.04 program (including GapCloser v1.12) (http://soap.genomics.org.cn/)^68^. Glimmer 3.0 (www.cbcb.umd.edu/software/glimmer) software was used to predict genes and Barrnap 0.4.2 and tRNA scan - SE v1.3.1 software to forecast the rRNA and tRNA of the genome. The protein sequences encoded by genes were blasted with each function database (KEGG, Nr, COG, String and GO).

### Gene network/pathway analysis

Predicted and annotated gene sequences were analyzed for similarity with the KEGG enzyme database followed by assignment of each gene into the KEGG (Kyoto Encyclopedia of Genes and Genomes) pathway chart. Based on individual analysis results of the KEGG pathways, integrated biochemical pathway maps were constructed which demonstrated characteristic physiological features in the metabolism of D5A. The existence of a certain pathway was then determined and integrated when component genes within the corresponding pathway had been completely identified.

### Phylogenetic analysis

The 16S rRNA gene sequences of *Klebsiella* sp. D5A were aligned with those of the publicly available *Klebsiella* genome sequences using the MAFFT (Multiple Alignment using Fast Fourier Transform) program based on 45 single copies of the homologous genes that were universally distributed in 16 analyzed *Klebsiella* genomes. The phylogenetic tree was constructed based on aligned concatenated sequences of these 45 genes using the bootstrap method available in RAxML (Randomized Axelerated Maximum Likelihood) which is a popular program for phylogenetic analysis of large datasets under maximum likelihood^69^.

### Data submission

The whole genome shotgun project of *Klebsiella* sp. D5A has been deposited at DDBJ/EMBL/GenBank under the accession LOAR00000000. The version described in this paper is version LOAR01000000. This strain has also been deposited in the CGMCC (China General Microbiological Culture Collection Center) under the accession number NO. 7248.

## Additional Information

**How to cite this article**: Liu, W. *et al*. Whole genome analysis of halotolerant and alkalotolerant plant growth-promoting rhizobacterium Klebsiella sp. D5A. *Sci. Rep.* **6**, 26710; doi: 10.1038/srep26710 (2016).

## Supplementary Material

Supplementary Information

## Figures and Tables

**Table 1:** General features of the *Klebsiella* sp. D5A genome.

Feature	Chromosome
Size (bp)	5,540,009
G + C content (%)	57.15
Number of CDSs	4999
Gene density	0.902 genes per kb
Average CDS length (bp)	944
tRNA	73
rRNA	4
Number of genes with assigned function	4129 (82.6%)
Number of genes without assigned function	870 (17.4%)

**Table 2:** COG functional categories of *Klebsiella* sp. D5A.

Type	Functional categories	COG
Information storage and processing	[A] RNA processing and modification	1
	[B] Chromatin structure and dynamics	1
	[J] Translation, ribosomal structure and biogenesis	179
	[K] Transcription	413
	[L] Replication, recombination and repair	164
Cellular processes and signaling	[D] Cell cycle control, cell division, chromosome partitioning	37
	[M] Cell wall/membrane/envelope biogenesis	242
	[N] Cell motility	29
	[O] Posttranslational modification, protein turnover, chaperones	153
	[T] Signal transduction mechanisms	141
	[U] Intracellular trafficking, secretion, and vesicular transport	78
	[V] Defense mechanisms	50
Metabolism	[C] Energy production and conversion	303
	[E] Amino acid transport and metabolism	500
	[F] Nucleotide transport and metabolism	94
	[G] Carbohydrate transport and metabolism	508
	[H] Coenzyme transport and metabolism	155
	[I] Lipid transport and metabolism	129
	[P] Inorganic ion transport and metabolism	362
	[Q] Secondary metabolites biosynthesis, transport and catabolism	106
Poorly characterized	[R] General function prediction only	297
	[S] Function unknown	397
Total		4339

**Table 3:** Biological and plant growth promotion properties and environmental tolerance of *Klebsiella* sp. D5A^15^.

S. No.	Attribute	D5A
1.	pH tolerance level	3.5–10.5
2.	Optimum pH for growth	4.0–10.0
3.	NaCl tolerance	up to 12%
4	Na_2_CO_3_ tolerance	25 mM
5.	IAA production	112 mg L^−1^
6.	Phosphate solubilization	131 mg L^−1^
7.	Siderophore production (A/Ar)	+
8.	Growth on N-free agar medium	Growth observed
