# Supplementary material for: Whole genome analysis of halotolerant and alkalotolerant plant growth-promoting rhizobacterium Klebsiella sp. D5A
Source: Sci Rep. 2016 May 24;6:26710. doi: 10.1038/srep26710 (PMC4877636; doi:10.1038/srep26710)
Supplement: Supplementary Information [file srep26710-s1.pdf]

# Whole genome analysis of halotolerant and alkalotolerant plant growth-promoting rhizobacterium Klebsiella sp. D5A

Wuxing Liu[1,*], Qingling Wang[1], Jinyu Hou[1], Tu Chen[2], Yongming Luo[2], Peter Christie[1]

Corresponding author
WX Liu (🖂) Key Laboratory of Soil Environment and Pollution Remediation, Institute of Soil Science, Chinese Academy of Sciences, Nanjing 210008, China.
E-mail: liuwuxin@issas.ac.cn

[1] Key Laboratory of Soil Environment and Pollution Remediation, Institute of Soil Science, Chinese Academy of Sciences, Nanjing 210008, China.
[2] Institute of Coastal Zone Research, Chinese Academy of Sciences, Yantai 264003, China.

Table S1 Genes related to PGP traits

| | Gene ID ORF | COG ID | Gene annotation | KO/Gene_ID | KEGG_GENE_NAME |
|---|---|---|---|---|---|
| Indole-3-acetic acid (IAA) biosynthesis | orf03176_1 | | nitrile hydratase, alpha subunit | K01721 | nthA |
| | orf03175_1 | | nitrile hydratase, beta subunit | K01721 | nthB |
| | orf03759_1 | COG3961 | indole-3-pyruvate decarboxylase | K04103 | ipdC |
| | orf03177_1 | COG0154 | amidase | K01426 | amiE |
| | orf00635_1 | COG1012 | aldehyde dehydrogenase | K00128 | E1.2.1.3 |
| Phosphate solubilization | orf03914_1 | _ | coenzyme PQQ synthesis protein D | K06138 | pqqD |
| | orf03915_1 | COG1235 | pyrroloquinoline quinone biosynthesis protein PqqB | K06136 | pqqB |
| | orf03916_1 | COG0535 | pyrroloquinoline quinone biosynthesis protein PqqE | K06139 | pqqE |
| | orf03917_1 | COG1025 | coenzyme PQQ biosynthesis protein PqqF | _ | pqqF |
| | orf03918_1 | COG5424 | pyrroloquinoline quinone biosynthesis protein PqqC | K06137 | pqqC |
| | orf01964_1 | COG4993 | quinoprotein glucose dehydrogenase | K00117 | gcd |
| Phosphate uptake | orf04595_1 | COG0226 | phosphate ABC transporter periplasmic substrate-binding protein PstS | K02040 | pstS |
| | orf04594_1 | COG0573 | phosphate transporter permease subunit PstC | K02037 | pstC |
| | orf04593_1 | COG0581 | phosphate transporter permease subunit PtsA | K02038 | pstA |
| | orf04592_1 | COG1117 | phosphate transporter subunit | K02036 | pstB |
| | orf01743_1 | COG0306 | low-affinity inorganic phosphate transporter | K16322 | pit |
| | orf04108_1 | COG3638 | phosphonate ABC transporter, ATP-binding protein | K02042 | phnC |
| | orf04109_1 | COG3221 | phosphonate ABC transporter, periplasmic phosphonate-binding protein | K05781 | phnD |
| | orf04111_1 | COG3639 | phosphonate ABC transporter, permease protein | K06163 | phnE1 |
| | orf04110_1 | COG3639 | phosphonate ABC transporter, permease protein | K06166 | phnE2 |

| | | | | | |
|---|---|---|---|---|---|
| Siderophore production | orf00726_1 | COG1028 | 2,3-dihydroxybenzoate-2,3-dehydrogenase | K00216 | entA |
| | orf00725_1 | COG3433;COG1535 | 2,3-dihydro-2,3-dihydroxybenzoate synthetase, isochroismatase | K01252 | entB, dhbB, vibB, mxcF |
| | orf00724_1 | COG1021 | enterobactin synthase subunit E | K02363 | entE, dhbE, vibE, mxcE |
| | orf00723_1 | COG1169 | isochorismate synthase EntC | K02361 | entC |
| | orf00721_1 | COG0477 | enterobactin exporter EntS | K08225 | entS |
| | orf00717_1 | COG1020;COG3319 | enterobactin synthase subunit F | K02364 | entF |
| | orf00713_1 | COG2977 | 4'-phosphopantetheinyl transferase EntD | K02362 | entD |
| Fe uptake | orf01388_1 | COG4771 | colicin I receptor FepA | K16089 | TC.FEV.OM2, fepA, iroN, cirA, pfeA, cfrA, hmuR |
| | orf00714_1 | COG4771 | outer membrane receptor/ferric enterobactin receptor FepA | K16089 | TC.FEV.OM2, fepA, iroN, cirA, pfeA, cfrA, hmuR |
| | orf02458_1 | COG4771 | outer membrane receptor/ferric enterobactin receptor FepA | K16089 | TC.FEV.OM2, fepA, iroN, cirA, pfeA, cfrA, hmuR |
| | orf04037_1 | COG4774 | catecholate siderophore receptor fiu | K16090 | fiu |
| | orf00543_1 | COG1629 | TonB-dependent receptor | | TC.FEV.OM3, tbpA, hemR, lbpA, hpuB, bhuR, hugA, hmbR |
| | orf00577_1 | _ | hypothetical protein(TonB-dependent siderophore receptor) | | TC.FEV.OM |
| | orf02635_1 | COG1629 | TonB-dependent siderophore receptor | | tonB |
| | orf02824_1 | COG1629 | TonB-dependent receptor | | ABC.FEV.P |
| | orf03828_1 | COG1629 | TonB-dependent receptor | | TC.FEV.OM |
| | orf04232_1 | COG1629 | ferric aerobactin receptor | K02014 | TC.FEV.OM |
| | orf03486_1 | COG1629 | ferrioxamine receptor foxA | K02014 | TC.FEV.OM |
| | orf01930_1 | COG1629 | ferrichrome outer membrane receptor | K02014 | TC.FEV.OM |
| | orf00816_1 | COG0735 | ferric uptake regulator | | fur |

| | | | | | |
|---|---|---|---|---|---|
| Fe transport | orf01825_1 | COG1918 | ferrous iron transport protein A | K04758 | feoA |
| | orf01824_1 | COG0370 | ferrous iron transport protein B | K04759 | feoB |
| | orf01823_1 | _ | hypothetical protein; May function as a transcriptional regulator that controls feoABC | K07490 | feoC |
| | orf00551_1 | COG1121 | iron transporter: fur regulated | K11607 | sitB |
| | orf00550_1 | COG1108 | iron ABC transporter, permease protein AfeC | K11605 | sitC |
| | orf00552_1 | COG0803 | iron transport system periplasmic binding component | K11604 | sitA |
| | orf00549_1 | COG1108 | iron ABC transporter, permease protein AfeD | K11606 | sitD |
| | orf03856_1 | COG1178 | ABC transporter, permease protein; Part of a binding-protein-dependent transport system. | | afuB, fbpB |
| | orf02844_1 | COG1120 | ABC transporter, iron chelate uptake transporter (FeCT) family, ATP-binding protein | K02013 | ABC.FEV.A |
| | orf02844_1 | COG1120 | ABC transporter, iron chelate uptake transporter (FeCT) family, ATP-binding protein | K02013 | ABC.FEV.A |
| | orf02843_1 | COG0609 | iron-siderophore ABC transporter permease | | TC.FEV.OM |
| | orf02843_1 | COG0609 | ABC transporter, iron chelate uptake transporter (FeCT) family, permease protein | K02015 | ABC.FEV.P |
| | orf02842_1 | COG0614 | ABC transporter, iron chelate uptake transporter (FeCT) family | | ABC.FEV.S |
| | orf02842_1 | COG0614 | ABC transporter, iron chelate uptake transporter (FeCT) family | K02016 | ABC.FEV.S |
| | orf02717_1 | COG0810 | transport protein TonB | K03832 | TC.FEV.OM |
| | orf02681_1 | COG1120 | iron ABC transporter, ATP-binding protein | K02013 | ABC.FEV.A |
| | orf02680_1 | COG0609 | iron ABC transporter, permease protein | K02015 | ABC.FEV.P |
| | orf02679_1 | COG0614 | iron ABC transporter, periplasmic iron-binding protein | K02016 | ABC.FEV.S |
| | orf02656_1 | COG1120 | iron chelate ABC transporter, ATP-binding protein | K02013 | ABC.FEV.A |
| | orf02655_1 | COG0609 | iron chelate ABC transporter, permease protein | K02015 | ABC.FEV.P |

| | | | | |
|---|---|---|---|---|
| orf02654_1 | COG0614 | iron chelate ABC transporter, periplasmic iron chelate-binding protein | K02016 | ABC.FEV.S |
| orf02634_1 | COG1120 | iron ABC transporter, ATP-binding protein | K02013 | ABC.FEV.A |
| orf02633_1 | COG0609 | iron ABC transporter, permease protein | K02015 | ABC.FEV.P |
| orf02632_1 | COG0609 | iron ABC transporter, permease protein | K02015 | ABC.FEV.P |
| orf02631_1 | COG0614 | iron ABC transporter, periplasmic iron-binding protein | K02016 | ABC.FEV.S |
| orf02617_1 | _ | _ | K02013 | ABC.FEV.A |
| orf02616_1 | _ | _ | K02015 | ABC.FEV.P |
| orf02615_1 | _ | _ | K02016 | ABC.FEV.S |
| orf02127_1 | COG4114 | ferric iron reductase involved in ferric hydroximate transport | K13255 | fhuF |
| orf01929_1 | COG1120 | iron-hydroxamate transporter ATP-binding subunit | K02013 | ABC.FEV.A |
| orf01928_1 | COG0614 | iron-hydroxamate transporter substrate-binding subunit | K02016 | ABC.FEV.S |
| orf01927_1 | COG0609 | iron-hydroxamate transporter permease subunit | K02015 | ABC.FEV.P |
| orf01099_1 | COG1178 | iron(III) ABC transporter, permease protein; | | afuB, fbpB |
| orf01098_1 | COG1840 | iron(III) ABC transporter, periplasmic iron(III)-binding protein | | afuA, fbpA |
| orf00722_1 | COG4592 | iron-enterobactin transporter periplasmic binding protein | K02016 | ABC.FEV.S |
| orf00720_1 | COG0609 | ferric enterobactin (enterochelin) transport | | ABC.FEV.P |
| orf00720_1 | COG0609 | ferric enterobactin (enterochelin) transport | K02015 | ABC.FEV.P |
| orf00719_1 | COG4779 | iron-enterobactin transporter permease | K02015 | ABC.FEV.P |
| orf00718_1 | COG1120 | iron-enterobactin transporter ATP-binding protein | K02013 | ABC.FEV.A |
| orf00541_1 | COG4558 | hemin ABC transporter, periplasmic hemin-binding protein | K02016 | ABC.FEV.S |
| orf00540_1 | COG0609 | hemin ABC transporter, permease protein | K02015 | ABC.FEV.P |
| orf00539_1 | COG4559 | hemin importer ATP-binding subunit | K02013 | ABC.FEV.A |
| orf00231_1 | COG0848 | biopolymer transport protein ExbD | K03559 | exbD |
| orf00230_1 | COG0811 | biopolymer transport protein ExbB | K03561 | exbB |

| | | | | | |
|---|---|---|---|---|---|
| Acetoin and 2, 3-butanediol synthesis | orf04628_1 | COG3978 | acetolactate synthase 2 regulatory subunit | K11258 | ilvM |
| | orf02240_1 | COG3962 | putative acetolactate synthase large subunit | K03336 | iolD |
| | orf02015_1 | COG0440 | acetolactate synthase 3 regulatory subunit | K01653 | E2.2.1.6S, ilvH, ilvN |
| | orf04551_1 | COG0440 | acetolactate synthase 1 regulatory subunit | K01653 | E2.2.1.6S, ilvH, ilvN |
| | orf02016_1 | COG0028 | acetolactate synthase 3 catalytic subunit | K01652 | E2.2.1.6L, ilvB, ilvG, ilvI |
| | orf02580_1 | COG0028 | acetolactate synthase | K01652 | E2.2.1.6L, ilvB, ilvG, ilvI |
| | orf00545_1 | COG0028 | acetolactate synthase catalytic subunit | K01652 | E2.2.1.6L, ilvB, ilvG, ilvI |
| | orf03057_1 | COG0028 | acetolactate synthase large subunit | K01576 | mdlC |
| | orf02581_1 | COG1028 | acetoin reductase | K03366 | butA, budC |
| | orf02579_1 | COG3527 | alpha-acetolactate decarboxylase | K01575 | E4.1.1.5, alsD |
| Antimicrobial compounds | | | | | |
| Phenazine production | orf03100_1 | COG0384 | phenazine biosynthesis protein PhzF family | | |
| 4-hydroxybenzoate production | orf02481_1 | COG3161 | chorismate pyruvate lyase | | ubiC |
| Chitinase production | orf04356_1 | COG3325 | putative chitinase II | K01183 | E3.2.1.14 |
| γ-aminobutyric acid (GABA) | orf01112_1 | COG1012 | putative succinate-semialdehyde dehydrogenase (NAD(P)(+)) | K00135 | gabD |
| | orf04143_1 | COG1012 | succinate-semialdehyde dehydrogenase I | K00135 | gabD |
| | orf02181_1 | COG1012 | aldehyde dehydrogenase (NAD) family protein | K00135 | gabD |
| | orf04144_1 | COG0160 | 4-aminobutyrate aminotransferase | K07250 | gabT |
| | orf04145_1 | COG1113 | gamma-aminobutyrate transporter | K11735 | gabP |
| Other PGPR fitness conferring genes | | | | | |
| | orf00281_1 | COG1166 | arginine decarboxylase | K01585 | E4.1.1.19S, speA |
| | orf00282_1 | COG0010 | agmatinase; Catalyzes the formation of putrescine from agmatine (By similarity) | K01480 | E3.5.3.11, speB |
| | orf01969_1 | COG0421 | spermidine synthase | K00797 | speE, SRM |

| | | | | | |
|---|---|---|---|---|---|
| Resistance to oxidative stress | | | | | |
| Peroxidase | orf03876_1 | COG2128;COG4950 | putative peroxidase | None | _ |
| | orf01147_1 | COG2837 | deferrochelatase/peroxidase EfeB | K16301 | efeB |
| | orf03730_1 | COG2837 | dyp-type peroxidase family protein | K07223 | K07223 |
| | orf04367_1 | COG0386 | glutathione peroxidase family protein | K00432 | E1.11.1.9 |
| | orf03910_1 | COG0376 | catalase/peroxidase HPI | K03782 | katG |
| | orf02698_1 | COG0386 | putative glutathione peroxidase | K00432 | E1.11.1.9 |
| | orf03689_1 | COG1225 | thioredoxin-dependent thiol peroxidase | K03564 | BCP, PRXQ, DOT5 |
| | orf03969_1 | COG2077 | thiol peroxidase | K11065 | tpx |
| Catalase | orf04050_1 | COG0753 | hydroperoxidase II; | K03781 | katE, CAT, catB, srpA |
| | orf03910_1 | COG0376 | catalase/peroxidase HPI | K03782 | katG |
| | orf04443_1 | COG3546 | putative manganese containing catalase | K07217 | K07217 |
| Superoxide dismutase | orf04808_1 | COG0605 | superoxide dismutase | K04564 | SOD2 |
| | orf04489_1 | COG0605 | superoxide dismutase | K04564 | SOD2 |
| | orf04818_1 | COG2032 | superoxide dismutase | K04565 | SOD1 |
| | orf00329_1 | _ | putative superoxide dismutase, iron | _ | _ |
| Glutathione S- | orf02200_1 | COG0346 | glutathione transferase, FosA | | _ |
| transferase | orf03847_1 | COG0625 | glutathione S-transferase domain protein | K11208 | yncG |
| | orf03255_1 | COG0625 | glutathione S-transferase | K00799 | GST, gst |
| | orf04138_1 | COG0625 | glutathione S-transferase family protein | K00799 | GST, gst |
| | orf00702_1 | COG0625 | putative S-transferase | _ | _ |
| | orf02440_1 | COG0625 | putative glutathione S-transferase | K00799 | GST, gst |
| | orf01661_1 | COG0625 | putative glutathione S-transferase | _ | _ |
| | orf00235_1 | COG0625 | putative glutathione S-transferase YghU | K11209 | yghU |
| | orf01497_1 | COG0625 | putative glutathione S-transferase | _ | _ |

| | | | | | |
|---|---|---|---|---|---|
| | orf00973_1 | COG0625 | glutathione S-transferase family protein | _ | _ |
| | orf01734_1 | COG0625 | glutathione S-transferase domain protein | K00799 | GST, gst |
| | orf01496_1 | COG0625 | glutathione S-transferase domain protein | _ | _ |
| Hydroperoxide | orf03015_1 | COG0450 | putative thiol - alkyl hydroperoxide reductase | K03386 | E1.11.1.15, PRDX, ahpC |
| | orf00764_1 | COG3634 | alkyl hydroperoxide reductase subunit F | K03387 | ahpF |
| | orf00763_1 | COG0450 | alkyl hydroperoxide reductase subunit C | K03386 | E1.11.1.15, PRDX, ahpC |
| Heat shock | orf00667_1 | COG0576 | heat shock protein GrpE; | K03687 | GRPE |
| | orf01089_1 | COG3785 | heat shock protein HspQ; | K11940 | hspQ |
| | orf01317_1 | COG0443 | DnaK domain protein | K04046 | yegD |
| | orf01832_1 | COG1188 | ribosome-associated heat shock protein HSP15 | K04762 | hslR |
| | orf02078_1 | COG0484 | chaperone protein DnaJ; Participates actively in the response to hyperosmotic | K03686 | dnaJ |
| | orf02079_1 | COG0443 | molecular chaperone DnaK; Acts as a chaperone | K04043 | dnaK |
| | orf02376_1 | COG0459 | chaperonin GroEL; | K04077 | groEL, HSPD1 |
| | orf02377_1 | COG0234 | co-chaperonin GroES | K04078 | groES, HSPE1 |
| | orf02914_1 | COG0326 | heat shock protein 90 | K04079 | htpG, HSP90A |
| | orf03065_1 | COG0071 | Heat shock protein20/alpha crystallin family protein | K04080 | ibpA |
| | orf03423_1 | COG3187 | heat shock protein HslJ | K03668 | hslJ |
| | orf03528_1 | COG0501 | heat shock protein HtpX | K03799 | htpX |
| | orf03650_1 | COG0443 | chaperone protein HscA | K04044 | hscA |
| | orf04566_1 | COG0071 | heat shock chaperone IbpB | K04081 | ibpB |
| | orf04567_1 | COG0071 | heat shock protein IbpA | K04080 | ibpA |
| Cold shock | orf01682_1 | COG1278 | major cold shock protein | K03704 | cspA |
| | orf00770_1 | COG1278 | cold shock protein CspE | K03704 | cspA |
| | orf04877_1 | COG1278 | 'cold-shock' DNA-binding domain protein | K03704 | cspA |

| | | | | | |
|---|---|---|---|---|---|
| | orf01018_1 | COG1278 | DNA replication inhibitor | K03704 | cspA |
| Rhodanese: catalysis of the detoxification of cyanide | orf02644_1 | COG0607 | rhodanese-like protein sulfurtransferase | None | _ |
| | orf00629_1 | COG0607 | rhodanese domain protein | None | _ |
| | orf01204_1 | COG0476;COG0607 | putative thiazole biosynthesis adenylyltransferase ThiF/rhodanese domain protein | K11996 | MOCS3, UBA4, moeB |
| | orf04193_1 | COG1054 | sulfurtransferase rhodanese | K07146 | K07146 |
| | orf03085_1 | COG0607 | rhodanese | None | _ |
| | orf01645_1 | COG0607 | rhodanese domain protein | None | _ |

Table S2 General genome features of the five studied *Klebsiella* genomes

| *Klebsiella* sp. | D5A | *variicola* At22 | *variicola* DX120 | *variicola* 342 | *pneumoniae*78578 |
|---|---|---|---|---|---|
| Genome size (bp) | 5540009 | 5458505 | 5718430 | 5920260 | 5694890 |
| G+C content (%) | 57.2 | 57.6 | 57.1 | 56.9 | 57.2 |
| Protein-coding genes | 4999 | 5113 | 5342 | 5554 | 5652 |
| No. of protein-coding genes with COGs | 4684 | 4979 | 5195 | 5369 | 5453 |
| rRNA genes | 4 | 25 | 25 | 25 | 25 |
| tRNA genes | 73 | 85 | 87 | 88 | 86 |
| Other RNA genes | _ | 2 | 1 | 1 | 1 |
| Plasmid | _ | _ | 2 | 2 | 5 |
| Accession number | LOAR00000000 | NC_013850.1 | NZ_CP009274.1 | NC_011283.1 | NC_009648.1 |

Table S3 Genes involved in the N cycle

| | Gene ID<br>ORF | COG ID | Gene annotation | KO/Gene_ID | KEGG_GENE_NAME |
|---|---|---|---|---|---|
| Nitrogenase complex | orf01255_1 | COG0674;COG1014;COG1013 | pyruvate-flavodoxin oxidoreductase | K03737 | nifJ |
| | orf01256_1 | COG1348 | nitrogenase iron protein | K02588 | nifH |
| | orf01257_1 | COG2710 | nitrogenase molybdenum-iron protein alpha chain | K02586 | nifD |
| | orf01258_1 | COG2710 | nitrogenase molybdenum-iron protein beta chain | K02591 | nifK |
| | orf01259_1 | _ | probable nitrogen fixation protein FixT | K02593 | nifT |
| | orf01260_1 | _ | NifY protein | None | nifY |
| | orf01261_1 | COG2710 | nitrogenase MoFe cofactor biosynthesis protein NifE | K02587 | nifE |
| | orf01262_1 | COG2710 | nitrogenase molybdenum-cofactor biosynthesis protein NifN | K02592 | nifN |
| | orf01263_1 | _ | NifX protein | K02596 | nifX |
| | orf01265_1 | COG1104 | cysteine desulfurase NifS | K04487 | nifS |
| | orf01266_1 | COG0119 | homocitrate synthase | K02594 | nifV |
| | orf01267_1 | _ | putative NifW protein | K02595 | nifW |
| | orf01268_1 | _ | putative NifZ protein | K02597 | nifZ |
| | orf01269_1 | COG0760 | nitrogen fixation protein NifM | K03769 | nifM |
| | orf01270_1 | COG0716 | flavodoxin FldA | K03839 | nifF |
| | orf01271_1 | COG2202 | nitrogen fixation negative regulator NifL | None | nifL |
| | orf01272_1 | COG3604 | Nif-specific regulatory protein | K02584 | nifA |
| | orf01273_1 | COG0535 | nitrogenase cofactor biosynthesis protein NifB | K02585 | nifB |
| | orf01274_1 | _ | nitrogen fixation protein NifQ | K15790 | nifQ |
| | orf03425_1 | COG0674;COG1014;COG1013 | putative pyruvate:ferredoxin (flavodoxin) oxidoreductase | K03737 | nifJ |
| | orf03647_1 | COG0822 | scaffold protein | K04488 | iscU, nifU |

| | | | | | |
|---|---|---|---|---|---|
| Nitrogenase | orf03772_1 | COG4657 | Na(+)-translocating NADH-quinone reductase subunit E | K03617 | rnfA |
| transport | orf03771_1 | COG2878 | electron transport complex protein RnfB; Required for nitrogen fixation. | K03616 | rnfB |
| | orf04836_1 | _ | electron transport complex protein rnfC | K03615 | rnfC |
| | orf04835_1 | COG4658 | electron transport complex protein RnfD; Required for nitrogen fixation. | K03614 | rnfD |
| | orf04833_1 | COG4660 | NADH-ubiquinone oxidoreductase | K03613 | rnfE |
| | orf04834_1 | COG4659 | electron transport complex protein RnfG | K03612 | rnfG |

Table S4 Genes involved in TCS

| orf | COG ID | Gene annotation | KO/Gene_ID | KEGG_GENE_NAME | Func Name |
|---|---|---|---|---|---|
| orf00171_1 | | | | | two-component system NarL family, sensor histidine kinase |
| orf00823_1 | COG0745 | KDP operon transcriptional regulatory protein KdpE | K07667 | kdpE | two-component system, OmpR family, KDP operon response regulator KdpE |
| orf00824_1 | COG2205 | sensor protein KdpD | K07646 | kdpD | two-component system, OmpR family, sensor histidine kinase KdpD |
| orf00995_1 | COG2205;COG0642 | sensor histidine kinase | _ | _ | |
| orf01318_1 | COG2205 | sensor histidine kinase | _ | _ | |
| orf01356_1 | COG3275 | putative two-compoent regulatory system sensor kinase | K02478 | K02478 | two-component system, LytT family, sensor kinase |
| orf01443_1 | COG0642;COG2198 | phosphotransfer intermediate protein in two-component regulatory system with RcsBC | K07676 | rcsD | two-component system, NarL family, sensor histidine kinase RcsD |
| orf01828_1 | COG0745 | osmolarity response regulator | K07659 | ompR | two-component system, OmpR family, phosphate regulon response regulator OmpR |
| orf01829_1 | COG0642 | osmolarity sensor protein | K07638 | envZ | two-component system, OmpR family, osmolarity sensor histidine kinase EnvZ |
| orf02044_1 | COG3290 | sensor histidine kinase | K07700 | dpiB, citA | two-component system, CitB family, cit operon sensor histidine kinase CitA |
| orf02097_1 | COG0745 | DNA-binding response regulator CreB | K07663 | creB | two-component system, OmpR family, catabolic regulation response regulator CreB |
| orf02415_1 | COG0642;COG0784 | sensor histidine | _ | _ | |

| | | | | | |
|---|---|---|---|---|---|
| | | kinase/response regulator | | | |
| orf02584_1 | COG0642 | sensor histidine protein kinase (RstA regulator) | _ | _ | |
| orf02748_1 | COG3850 | nitrate/nitrite sensor protein NarX | K07673 | narX | two-component system, NarL family, nitrate/nitrite sensor histidine kinase NarX |
| orf02749_1 | COG2197 | transcriptional regulator NarL | K07684 | narL | two-component system, NarL family, nitrate/nitrite response regulator NarL |
| orf03182_1 | COG0642 | sensor histidine kinase | _ | _ | |
| orf03306_1 | COG3290 | sensory histidine kinase DcuS | K07701 | dcuS | two-component system, CitB family, sensor histidine kinase DcuS |
| orf03631_1 | COG0642 | putative 2-component sensor protein | K07711 | glrK, qseE | two-component system, NtrC family, sensor histidine kinase GlrK |
| orf03763_1 | COG3275 | sensor histidine kinase | K02478 | K02478 | two-component system, LytT family, sensor kinase |
| orf03902_1 | COG3662 | hypothetical protein/ histidine kinase | _ | _ | _ |
| orf03922_1 | COG0642 | sensor histidine kinase | _ | _ | _ |
| orf04277_1 | COG2200;COG2203 | histidine kinase/ cyclic diguanylate phosphodiesterase (EAL) domain protein | _ | _ | _ |
| orf04487_1 | COG0642 | two-component sensor protein | K07640 | cpxA | two-component system, OmpR family, sensor histidine kinase CpxA |
| orf04549_1 | COG3851 | sensory histidine kinase UhpB | K07675 | uhpB | two-component system, NarL family, sensor histidine kinase UhpB |

Table S5 Genes involved in salt tolerance

| | Gene ID | COG ID | Gene annotation | KO/Gene_ID | KEGG_GENE_NAME |
|---|---|---|---|---|---|
| | ORF | | | | |
| Glycine-betaine synthesis | orf00698_1 | COG1292 | choline transport protein BetT | K02168 | betT, betS |
| | orf00696_1 | COG1012 | betaine aldehyde dehydrogenase | K00130 | betB, gbsA |
| | orf00695_1 | COG2303 | choline dehydrogenase | K00108 | betA, CHDH |
| | orf00697_1 | COG1309 | transcriptional regulator BetI | K02167 | betI |
| | orf02401_1 | COG0477 | proline/glycine betaine transporter | K03762 | proP |
| | orf00615_1 | COG4176 | glycine betaine transporter membrane protein | K02001 | proW |
| | orf00616_1 | COG4175 | glycine betaine/L-proline ABC transporter, ATP-binding protein | K02000 | proV |
| | orf00614_1 | COG2113 | glycine betaine transporter periplasmic subunit | K02002 | proX |
| | orf01141_1 | COG0591 | sodium/proline symporter | K11928 | putP |
| | orf01114_1 | COG1292 | putative transporter | K03451 | TC.BCT |
| Trehalose metabolism | orf03896_1 | COG0366 | putative trehalose synthase | K05343 | treS |
| | orf01732_1 | COG1626 | trehalase | K01194 | treA, treF |
| | orf03581_1 | COG0380 | trehalose-6-phosphate synthase | K00697 | otsA |
| | orf03582_1 | COG1877 | trehalose-6-phosphate phosphatase | K01087 | otsB |
| | orf02262_1 | COG1609 | trehalose repressor | K03485 | treR1, treR |
| Genes involved in $Na^{+}$ and $K^{+}$ transport | orf00035_1 | COG0471 | transporter, divalent anion:Na+ symporter (DASS) family | None | _ |
| | orf00070_1 | COG0530 | putative calcium/sodium:proton antiporter | K07301 | yrbG |
| | orf01209_1 | COG0025 | Na+/H+ antiporter | K03316 | TC.CPA1 |
| | orf01488_1 | COG0471 | transporter, divalent anion:Na+ symporter (DASS) family | None | _ |
| | orf02077_1 | COG3004 | pH dependent Na+/H antiporter | K03313 | nhaA |
| | orf02438_1 | COG0025 | Na+/H+ antiporter | K03316 | TC.CPA1 |
| | orf02493_1 | COG0385 | Na+ symporter, BASS family | K03453 | TC.BASS |
| | orf03496_1 | COG3067 | Sodium/proton antiporter; Na (+)/H (+) antiporter t | K03314 | nhaB |

| | | | | |
|---|---|---|---|---|
| orf04048_1 | COG1823 | transporter, dicarboxylate/amino acid:cation (Na+ or H+) symporter (DAACS) family | K06956 | K06956 |
| orf00827_1 | COG2060 | potassium-transporting ATPase subunit A | K01546 | kdpA |
| orf00826_1 | COG2216 | potassium-transporting ATPase subunit B | K01547 | kdpB |
| orf00825_1 | | potassium-transporting ATPase subunit C | K01548 | kdpC |
| orf02076_1 | COG0583 | transcriptional activator NhaR | K03717 | nhaR |
| orf02724_1 | COG1226 | transporter, cation channel family | K10716 | kch, trkA, mthK, pch |
| orf02910_1 | COG1226;COG4651 | putative cation:proton antiport protein | K03455 | TC.KEF |
| orf02923_1 | COG3264 | potassium efflux protein KefA | K05802 | kefA, bspA, aefA |
| orf03491_1 | COG3263 | potassium/proton antiporter | K11105 | cvrA, nhaP2 |
| orf03883_1 | COG0221 | inorganic diphosphatase /potassium-transporting ATPase subunit C | K01507 | ppa |
| orf04615_1 | COG3158 | potassium transport protein Kup | K03549 | kup |
| orf04699_1 | COG0168 | potassium transporter | K03498 | trkH |
| orf04935_1 | COG0569 | potassium transporter peripheral membrane component | K03499 | trkA |

Table S6 Genes involved in pH wide adaptation

| | Gene ID<br>ORF | COG ID | Gene annotation | KO/Gene_ID | KEGG_GENE_NAME |
|---|---|---|---|---|---|
| Acid expressed | orf00827_1 | COG2060 | potassium-transporting ATPase subunit A | K01546 | kdpA |
| | orf00826_1 | COG2216 | potassium-transporting ATPase subunit B | K01547 | kdpB |
| | orf00825_1 | COG2156 | potassium-transporting ATPase subunit C | K01548 | kdpC |
| | orf00824_1 | COG2205 | sensor protein KdpD | K07646 | kdpD |
| | orf00823_1 | COG0745 | KDP operon transcriptional regulatory protein KdpE | K07667 | kdpE |
| | orf04595_1 | COG0226 | phosphate ABC transporter periplasmic substrate-binding protein PstS | K02040 | pstS |
| | orf04594_1 | COG0573 | phosphate transporter permease subunit PstC | K02037 | pstC |
| | orf04593_1 | COG0581 | phosphate transporter permease subunit PtsA | K02038 | pstA |
| | orf04592_1 | COG1117 | phosphate transporter subunit | K02036 | pstB |
| | orf02866_1 | COG1982 | lysine decarboxylase 1 | K01582 | ldcC, cadA |
| | orf01895_1 | COG1982 | lysine decarboxylase 2, constitutive | K01582 | ldcC, cadA |
| | orf02966_1 | COG0776 | transcriptional regulator HU subunit beta | K03530 | hupB |
| | orf04859_1 | COG0776 | transcriptional regulator HU subunit alpha | K05787 | hupA |
| | orf00942_1 | COG0783 | DNA starvation/stationary phase protection protein Dps | K04047 | dps |
| | orf01957_1 | COG0853 | aspartate alpha-decarboxylase | K01579 | panD |
| | orf02969_1 | COG1219 | ATP-dependent protease ATP-binding subunit ClpX | K03544 | clpX, CLPX |
| | orf02970_1 | COG0740 | ATP-dependent Clp protease proteolytic subunit | K01358 | clpP, CLPP |
| | orf02477_1 | COG1974 | LexA repressor; Represses a number of genes involved in the response to DNA damage | K01356 | lexA |
| | orf00190_1 | COG0804 | urease subunit alpha | K01428 | ureC |
| | orf00191_1 | COG0832 | urease subunit beta | K01429 | ureB |
| | orf00192_1 | COG0831 | urease subunit gamma | K01430 | ureA |

| | | | | | |
|---|---|---|---|---|---|
| | orf00189_1 | COG2371 | urease accessory protein UreE | K03187 | ureE |
| | orf00188_1 | COG0830 | urease accessory protein UreF | K03188 | ureF |
| | orf00187_1 | COG0378 | urease accessory protein UreG | K03189 | ureG |
| | orf00193_1 | COG0829 | urease accessory protein UreD | K03190 | ureD, ureH |
| | orf02079_1 | COG0443 | molecular chaperone DnaK; Acts as a chaperone (By similarity) | K04043 | dnaK |
| | orf02376_1 | COG0459 | chaperonin GroEL | K04077 | groEL, HSPD1 |
| | orf00173_1 | | HdeB family protein | | |
| Alkaline expressed | orf03496_1 | COG3067 | sodium/proton antiporter; Na(+)/H(+) antiporter | K03314 | nhaB |
| | orf03491_1 | COG3263 | potassium/proton antiporter; Participates in control of cell volume in low-osmolarity condition | K11105 | cvrA, nhaP2 |
| | orf02910_1 | COG1226;COG4651 | putative cation:proton antiport protein | K03455 | TC.KEF |
| | orf02761_1 | COG0387 | calcium/sodium:proton antiporter | K07300 | chaA, CAX |
| | orf02438_1 | COG0025 | Na+/H+ antiporter | K03316 | TC.CPA1 |
| | orf02077_1 | COG3004 | pH dependent Na+/H antiporter | K03313 | nhaA |
| | orf02076_1 | COG0583 | transcriptional activator NhaR | K03717 | nhaR |
| | orf01209_1 | COG0025 | Na+/H+ antiporter | K03316 | TC.CPA1 |
| | orf00977_1 | COG0477 | multidrug translocase MdfA | K08160 | cmr, mdfA |
| | orf04606_1 | COG0356 | ATP synthase subunit A; Key component of the proton channel; | K02108 | ATPF0A, atpB |
| | orf04604_1 | COG0711 | F0F1 ATP synthase subunit B | K02109 | ATPF0B, atpF |
| | orf04605_1 | COG0636 | F0F1 ATP synthase subunit C | K02110 | ATPF0C, atpE |
| | orf04602_1 | COG0056 | F0F1 ATP synthase subunit alpha | K02111 | ATPF1A, atpA |
| | orf04600_1 | COG0055 | F0F1 ATP synthase subunit beta | K02112 | ATPF1B, atpD |
| | orf04603_1 | COG0712 | F0F1 ATP synthase subunit delta | K02113 | ATPF1D, atpH |
| | orf04599_1 | COG0355 | F0F1 ATP synthase subunit epsilon | K02114 | ATPF1E, atpC |
| | orf04601_1 | COG0224 | F0F1 ATP synthase subunit gamma | K02115 | ATPF1G, atpG |

| | | | | | |
|---|---|---|---|---|---|
| | orf04607_1 | COG3312 | F0F1 ATP synthase subunit I | K02116 | atpI |
| | orf02722_1 | COG1502 | cardiolipin synthetase | K06131 | cls |
| | orf02485_1 | COG2182 | maltose ABC transporter periplasmic protein | K10108 | malE |
| | orf03436_1 | COG2885 | OmpA domain protein | None | _ |
| | orf04704_1 | COG2885 | OmpA domain protein | None | _ |
| | orf00944_1 | COG3637 | outer membrane protein X | K11934 | ompX |
| | orf03514_1 | COG1760 | L-serine deaminase | K01752 | sdaA |
| | orf00491_1 | COG1760 | L-serine ammonia-lyase 2 | K01752 | sdaA |

Table S7 Genes involved in degradation of aromatic compounds

| Gene ID ORF | COG ID | Gene annotation | KO/Gene_ID | KEGG_GENE_NAME |
|---|---|---|---|---|
| orf02133_1 | COG0654 | 4-hydroxybenzoate 3-monooxygenase | K00481 | pobA |
| orf02132_1 | COG2207 | transcriptional regulator PobR | None | _ |
| orf03864_1 | COG4638 | 2-chlorobenzoate 1,2-dioxygenase, alpha subunit | K05549 | benA-xylX |
| orf03865_1 | COG5517 | 3-phenylpropionate dioxygenase beta subunit | K05550 | benB-xylY |
| orf03800_1 | COG3135 | benzoate transporter family protein | K05782 | benE |
| orf03867_1 | COG1028 | 1,6-dihydroxycyclohexa-2,4-diene-1-carboxylate dehydrogenase | K05783 | benD-xylL |
| orf03866_1 | COG0543 | 2-chlorobenzoate 1,2-dioxygenase, electron transfer subunit | K05784 | benC-xylZ |
| orf03863_1 | COG3485 | catechol 1,2-dioxygenase | K03381 | catA |
| orf03861_1 | COG4948 | muconate cycloisomerase | K01856 | catB |
| orf03862_1 | COG4829 | muconolactone delta-isomerase | K03464 | catC |
| orf03315_1 | COG0015 | 3-carboxy-cis,cis-muconate cycloisomerase | K01857 | pcaB |
| orf00326_1 | COG0599 | carboxymuconolactone decarboxylase family protein | K01607 | pcaC |
| orf03120_1 | COG0599 | carboxymuconolactone decarboxylase family protein | K01607 | pcaC |
| orf03313_1 | COG0599 | 4-carboxymuconolactone decarboxylase | K01607 | pcaC |
| orf04377_1 | _ | hypothetical protein | K01607 | pcaC |
| orf04471_1 | COG0599 | carboxymuconolactone decarboxylase family protein | K01607 | pcaC |
| orf03314_1 | COG0596 | 3-oxoadipate enol-lactonase | K01055 | pcaD |
| orf03787_1 | COG3485 | protocatechuate 3,4-dioxygenase, alpha subunit | K00448 | pcaG |
| orf03788_1 | COG3485 | protocatechuate 3,4-dioxygenase, beta subunit | K00449 | pcaH |
| orf03318_1 | COG1788 | 3-oxoadipate CoA-transferase, subunit A | K01031 | pcaI |
| orf03317_1 | COG2057 | 3-oxoadipate CoA-transferase, subunit B | K01032 | pcaJ |
| orf01684_1 | COG0477 | 4-hydroxybenzoate transporter | K08195 | pcaK |
| orf02987_1 | COG0477 | 4-hydroxybenzoate transporter | K08195 | pcaK |

| | | | | |
|---|---|---|---|---|
| orf02981_1 | COG1414 | transcriptional regulator, IclR family | K02624 | pcaR |
| orf03319_1 | COG1414 | Pca regulon regulatory protein | K02624 | pcaR |
| orf03860_1 | COG1414 | transcriptional regulator, IclR family | K02624 | pcaR |
| orf02646_1 | COG0477 | putative 3-hydroxyphenylpropionic transporter MhpT | K05819 | mhpT |
| orf02653_1 | COG1414 | DNA-binding transcriptional activator MhpR | K05818 | mhpR |
| orf02650_1 | COG0596 | 2-hydroxy-6-ketonona-2,4-dienedioic acid hydrolase | K05714 | mhpC |
| orf02651_1 | _ | 3-(2,3-dihydroxyphenyl)propionate dioxygenase | K05713 | mhpB |
| orf02652_1 | COG0654 | 3-(3-hydroxyphenyl)propionate hydroxylase | K05712 | mhpA |
| orf02648_1 | COG4569 | acetaldehyde dehydrogenase; Catalyzes the conversion of acetaldehyde to acetyl-CoA | K04073 | mhpF |
| orf02649_1 | COG3971 | 2-keto-4-pentenoate hydratase | K02554 | mhpD |
| orf02647_1 | COG0119 | 4-hydroxy-2-oxovalerate aldolase | K01666 | mhpE |

Table S8 Genes involved in the sulfur cycle

| | Gene ID | COG ID | Gene annotation | KO/Gene_ID | KEGG_GENE_NAME |
|---|---|---|---|---|---|
| | ORF | | | | |
| Thiosulfate or sulfate transporter and convert-related genes | orf00517_1 | COG0583 | transcriptional regulator CysB | K13634 | cysB |
| | orf01167_1 | COG0031 | putative cysteine synthase B | K12339 | cysM |
| | orf03744_1 | COG0031 | cysteine synthase B | K12339 | cysM |
| | orf01649_1 | COG0765 | amino acid ABC transporter, permease protein | K10009 | ABC.CYST.P |
| | orf03733_1 | COG2981 | putative sulfate transport protein CysZ | K06203 | cysZ |
| | orf00512_1 | COG4150 | thiosulfate transporter subunit | K02048 | cysP, sbp |
| | orf02887_1 | COG1613 | sulfate transporter subunit | K02048 | cysP, sbp |
| | orf00513_1 | COG4208 | sulfate/thiosulfate transporter permease subunit | K02047 | cysW |
| | orf01168_1 | COG0555 | sulfate/thiosulfate transporter subunit | K02046 | cysU |
| | orf00514_1 | COG1118 | sulfate/thiosulfate transporter subunit | K02045 | cysA |
| | orf02959_1 | COG0031 | cysteine synthase A | K01738 | cysK |
| | orf04011_1 | COG0031 | pyridoxal-phosphate dependent enzyme | K01738 | cysK |
| | orf03731_1 | COG0175 | sulfate adenylyltransferase subunit 2 | K00957 | cysD |
| | orf01591_1 | COG2895 | sulfate adenylyltransferase subunit 1 | K00956 | cysN |
| | orf03734_1 | COG0529 | adenylylsulfate kinase | K00860 | cysC |
| | orf02306_1 | COG0175 | phosphoadenosine phosphosulfate reductase | K00390 | cysH |
| | orf03735_1 | COG0155 | sulfite reductase subunit beta | K00381 | cysI |
| | orf01207_1 | COG0369 | sulfite reductase subunit alpha | K00380 | cysJ |
| Sulfonate or alkanesulfonate related genes | orf01069_1 | COG1116 | aliphatic sulfonates transport ATP-binding subunit | K15555 | ssuB |
| | orf01066_1 | COG1116 | putative sulfonate ABC transporter, ATP-binding protein | K15555 | ssuB |
| | orf03957_1 | COG0600 | alkanesulfonate transporter permease subunit | K15554 | ssuC |
| | orf02641_1 | COG0600 | putative sulfonate ABC transporter, permease protein | K15554 | ssuC |
| | orf03958_1 | COG0715 | putative ABC-type transport protein | K15553 | ssuA |

| | | | | | |
|---|---|---|---|---|---|
| | orf02639_1 | COG0715 | aliphatic sulfonates ABC transporter family, periplasmic substrate-binding protein | K15553 | ssuA |
| | orf01068_1 | COG0715 | aliphatic sulfonates ABC transporter, periplasmic sulfonate-binding protein | K15553 | ssuA |
| | orf01067_1 | COG0715 | putative sulfonate ABC transporter, sulfonate-binding protein | K15553 | ssuA |
| | orf01065_1 | COG2141 | alkanesulfonate monooxygenase | K04091 | ssuD |
| | orf03959_1 | COG0431 | NAD(P)H-dependent FMN reductase | K00299 | ssuE |
| Taurine transporter | orf03341_1 | COG4521 | taurine ABC transporter, periplasmic taurine-binding protein | K15551 | tauA |
| | orf04083_1 | COG0600 | taurine transporter subunit; Part of a binding-protein-dependent transport system | K15552 | tauC |
| | orf04084_1 | COG4525 | taurine transporter ATP-binding subunit | K10831 | tauB |
| | orf04085_1 | COG2175 | taurine dioxygenase | K03119 | tauD |

Table S9 Genes involved in secretion systems

| | Gene ID ORF | COG ID | Gene annotation | KO/Gene_ID | KEGG_GENE_NAME |
|---|---|---|---|---|---|
| tat | orf00773_1 | COG1826 | twin arginine translocase protein E | K03425 | tatE |
| | orf02114_1 | COG0084 | hydrolase, TatD family | K03424 | tatD |
| | orf04222_1 | COG0084 | putative metallodependent hydrolase | K03424 | tatD |
| | orf04688_1 | COG1826 | twin arginine translocase protein A | K03116 | tatA |
| | orf04690_1 | COG0805 | twin-arginine protein translocation system subunit TatC | K03118 | tatC |
| | orf04689_1 | COG1826 | sec-independent translocase | K03117 | tatB |
| | orf04691_1 | COG0084 | DNase TatD | K03424 | tatD |
| sec | orf04837_1 | COG0690 | preprotein translocase subunit SecE | K03073 | secE |
| | orf00091_1 | COG1314 | preprotein translocase subunit SecG | K03075 | secG |
| | orf04927_1 | COG0201 | preprotein translocase subunit SecY; Involved in protein export (By similarity) | K03076 | secY |
| | orf03010_1 | COG0342 | preprotein translocase subunit SecD | K03072 | secD |
| | orf03617_1 | COG3086 | SoxR reducing system protein RseC | K03803 | rseC |
| | orf00217_1 | COG0642 | sensor protein QseC | K07645 | qseC |
| | orf03009_1 | COG0341 | preprotein translocase subunit SecF | K03074 | secF |
| | orf01996_1 | _ | SecA regulator SecM | K13301 | secM |
| | orf01647_1 | COG1952 | preprotein translocase subunit SecB | K03071 | secB |
| | orf01995_1 | COG0653 | preprotein translocase subunit SecA | K03070 | secA |
| | orf02973_1 | COG3056 | hypothetical protein | K07286 | yajG |
| | orf03011_1 | COG1862 | preprotein translocase subunit YajC | K03210 | yajC |
| | orf03167_1 | COG2188 | putative transcriptional regulator | K03482 | yidP |
| | orf04561_1 | COG2149 | hypothetical protein | K00389 | yidH |
| | orf04579_1 | COG0706 | putative inner membrane protein translocase component YidC | K03217 | yidC, spoIIIJ, OXA1 |
| | orf04564_1 | COG2188 | transcriptional regulator, GntR family | K03482 | yidP |

| | | | | | |
|---|---|---|---|---|---|
| | orf01521_1 | COG2188 | transcriptional regulator, GntR family | K03482 | yidP |
| | orf00087_1 | COG0293 | 23S rRNA methyltransferase J | K02427 | rlmE, rrmJ, ftsJ |
| | orf02010_1 | COG0768 | peptidoglycan synthetase FtsI | K03587 | ftsI |
| | orf02001_1 | COG1589 | cell division protein FtsQ | K03589 | ftsQ |
| | orf01774_1 | COG2884 | cell division protein FtsE | K09812 | ftsE |
| | orf03523_1 | COG0768 | putative peptidoglycan synthetase FtsI | K03587 | ftsI |
| | orf01775_1 | COG2177 | cell division protein FtsX | K09811 | ftsX |
| | orf00521_1 | COG2919 | cell division protein FtsB; Required for the cell division process (By similarity) | K05589 | ftsB |
| | orf02005_1 | COG0772 | cell division protein FtsW | K03588 | ftsW, spoVE |
| | orf01773_1 | COG0552 | cell division protein FtsY | K03110 | ftsY |
| | orf01999_1 | COG0206 | cell division protein FtsZ; | K03531 | ftsZ |
| | orf00088_1 | COG0465 | ATP-dependent metalloprotease | K03798 | ftsH, hflB |
| | orf02000_1 | COG0849 | cell division protein FtsA | K03590 | ftsA |
| | orf01027_1 | COG1674 | cell division protein | K03466 | ftsK, spoIIIE |
| | orf02011_1 | COG3116 | cell division protein FtsL | K03586 | ftsL |
| | orf04478_1 | COG3087 | cell division protein FtsN | K03591 | ftsN |
| | orf00670_1 | COG0541 | signal recognition particle protein | K03106 | SRP54, ffh |
| | orf03619_1 | COG0681 | signal peptidase I | K03100 | lepB |
| | orf02072_1 | COG0597 | lipoprotein signal peptidase | K03101 | lspA |
| Type I | orf00209_1 | COG1538 | outer membrane channel protein | K12340 | tolC |
| | orf00413_1 | _ | hypothetical protein | None | _ |
| | orf01558_1 | COG1538 | putative outer membrane efflux protein | None | _ |
| | orf00339_1 | COG1538 | putative outer membrane efflux protein MdtP | K15550 | mdtP |
| Type II | orf01935_1 | COG3031 | pullulanase secretion envelope protein PulC | K02452 | gspC |
| | orf01940_1 | _ | pullulanase secretion protein PulH | K02457 | gspH |
| | orf01941_1 | COG2165 | pullulanase secretion protein PulI | K02458 | gspI |

| | | | | | |
|---|---|---|---|---|---|
| | orf01936_1 | COG1450 | pullulanase secretion envelope protein PulD; | K02453 | gspD |
| | orf01942_1 | COG4795 | pullulanase secretion protein PulJ | K02459 | gspJ |
| | orf01938_1 | COG1459 | pullulanase secretion protein PulF | K02455 | gspF |
| | orf01939_1 | COG2165 | pullulanase G protein; | K02456 | gspG |
| | orf01947_1 | COG1989 | pullulanase secretion protein PulO; | K02464 | gspO |
| | orf01943_1 | COG3156 | pullulanase secretion protein PulK | K02460 | gspK |
| | orf01933_1 | _ | pullulanase-specific type II secretion system component B | K02451 | gspB |
| | orf01932_1 | _ | pullulanase secretion protein PulS | K02465 | gspS |
| | orf00238_1 | COG0754 | bifunctional glutathionylspermidine amidase/glutathionylspermidine synthetase | K01460 | gsp |
| | orf01945_1 | COG3149 | pullulanase secretion protein PulM | K02462 | gspM |
| | orf01937_1 | COG2804 | pullulanase secretion protein PulE | K02454 | gspE |
| | orf01946_1 | _ | pullulanase secretion protein PulN | K02463 | gspN |
| | orf01944_1 | COG3297 | pullulanase secretion protein PulL | K02461 | gspL |
| Type III | orf03073_1 | COG3395 | YgbK domain protein | None | _ |
| | orf03105_1 | COG3395 | hypothetical protein | None | _ |
| | orf04783_1 | _ | type III restriction enzyme, res subunit | None | _ |
| | orf01416_1 | COG1061 | putative helicase | K17677 | IRC3 |
| Type V | orf03618_1 | COG0481 | GTP-binding protein LepA | K03596 | lepA |
| | orf03619_1 | COG0681 | signal peptidase I | K03100 | lepB |
| Type VI | orf04715_1 | COG3521 | hypothetical protein | K11906 | vasD, lip |
| | orf01541_1 | COG3521 | hypothetical protein | K11906 | vasD, lip |
| | orf04706_1 | COG0542 | type VI secretion ATPase, ClpV1 family | K11907 | vasG, clpV |
| | orf03437_1 | COG3455 | type IV/VI secretion system protein, DotU family | K11892 | impK, ompA, vasF, dotU |
| | orf04712_1 | COG3515 | ImpA domain-containing protein | K11910 | vasJ |
| | orf02779_1 | COG3518 | type VI secretion system lysozyme-related protein | K11905 | K11905 |
| | orf04714_1 | COG3520 | hypothetical protein | K11895 | impH, vasB |

| | | | | |
|---|---|---|---|---|
| orf04705_1 | COG3157 | hypothetical protein | K11903 | K11903, hcp |
| orf02781_1 | COG3520 | type VI secretion protein, VC_A0111 family | K11895 | impH, vasB |
| orf04713_1 | COG3519 | hypothetical protein | K11896 | impG, vasA |
| orf04703_1 | COG3455 | type IV/VI secretion system protein, DotU family | K11892 | impK, ompA, vasF, dotU |
| orf04979_1 | _ | _ | K11906 | vasD, lip |
| orf04702_1 | COG3522 | hypothetical protein | K11893 | impJ, vasE |
| orf02780_1 | COG3521 | type VI secretion lipoprotein, VC_A0113 family | K11906 | vasD, lip |
| orf02778_1 | COG3515 | ImpA domain protein | K11911 | vasL |
| orf04990_1 | COG3522 | hypothetical protein | K11893 | impJ, vasE |
| orf04711_1 | COG3523 | hypothetical protein | K11891 | impL, vasK, icmF |
| orf02783_1 | COG3523 | ImcF-related family protein | K11891 | impL, vasK, icmF |
| orf04989_1 | COG3455 | hypothetical protein | K11892 | impK, ompA, vasF, dotU |
